# Complicated enteroenteric intestinal fistula due to Crohn’s disease: a rare case report

**DOI:** 10.1093/jscr/rjaf250

**Published:** 2025-07-27

**Authors:** Muhanned Alkhatıb, Hilmi Bozkurt, Cumhur Ozcan, Hasan Hüsnü Yüksek, Enver Reyhan

**Affiliations:** Department of General Surgery, Faculty of Medicine, Mersin University, Ciftlikkoy Campus, Yenisehir, Mersin 33343, Turkey; Department of General Surgery, Faculty of Medicine, Mersin University, Ciftlikkoy Campus, Yenisehir, Mersin 33343, Turkey; Department of General Surgery, Faculty of Medicine, Mersin University, Ciftlikkoy Campus, Yenisehir, Mersin 33343, Turkey; Department of Radiology, Faculty of Medicine, Mersin University, Ciftlikkoy Campus, Yenisehir, Mersin 33343, Turkey; Department of General Surgery, Faculty of Medicine, Mersin University, Ciftlikkoy Campus, Yenisehir, Mersin 33343, Turkey

**Keywords:** Crohn’s disease, intestinal tuberculosis, complicated fistula, surgical management, diagnostic challenges

## Abstract

Crohn’s disease can lead to complications such as fistula formation, with ileosigmoid fistulas being common. This case report presents a 54-year-old male with Crohn’s disease, who had abdominal pain, diarrhea, and weight loss. He had a history of miliary tuberculosis, which complicated the diagnostic process. Imaging revealed fistulas and thickening in the terminal ileum, while surgery included ileocecectomy, fistulectomy, and repair of affected areas. The patient recovered without complications. This case highlights the diagnostic challenges when Crohn’s disease coexists with infectious diseases like tuberculosis and underscores the importance of accurate imaging and a multidisciplinary approach for diagnosis and treatment.

## Introduction

In Crohn’s disease, fistula development has been reported in 20%–40% of different studies [1–4]. Ileosigmoid fistulas are a complication of Crohn’s disease, which often involves the terminal ileum, and are the most common fistulas seen between two intestinal segments. Enteroenteric, gastrocolic, duodenocolic, enterovesical, rectovaginal, and perianal fistulas are other possible potential complications of Crohn’s disease. These fistulas, which are quite problematic to treat, have an average mortality rate of 7% [5]. Although biologic agents are effective against fistulizing Crohn’s disease in about half of patients [6], refractory fistulas require resection of the involved bowel segment and suturing of the fistula opening to the adjacent organ. We aimed to present the surgical diagnosis, treatment, and follow-up process of our case with internal fistulas and related complications due to Crohn’s disease.

## Case report

**Figure 1.**
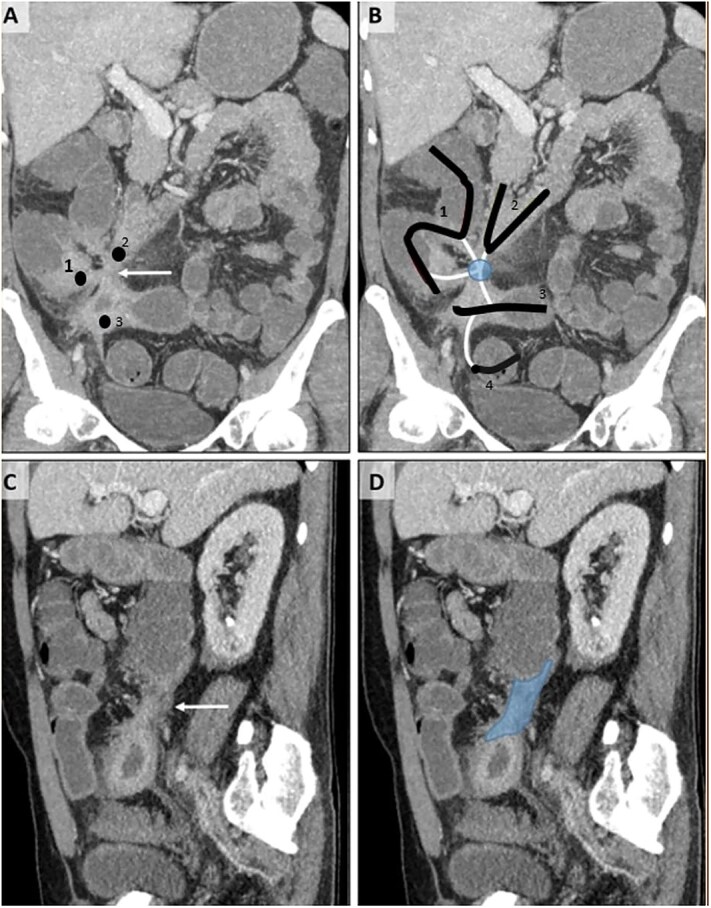
CT enterography examination, coronal (A, B) and sagittal (C, D) MIP (maximum intensity projection) images.Thickening and increased contrast enhancement are observed in the walls of the 2nd–3rd parts of the duodenum (2), terminal ileum (3), and cecum (1) (A). The relationship of these structures, whose lumens are indicated by the same numbers, with the tract-like structures (white lines) of the sigmoid colon (4) — fistulization (circle) — is shown (B). In the sagittal images (C, D), a fistula tract (D, dark area) with a thick wall (C, white arrow) is observed.

**Figure 2.**
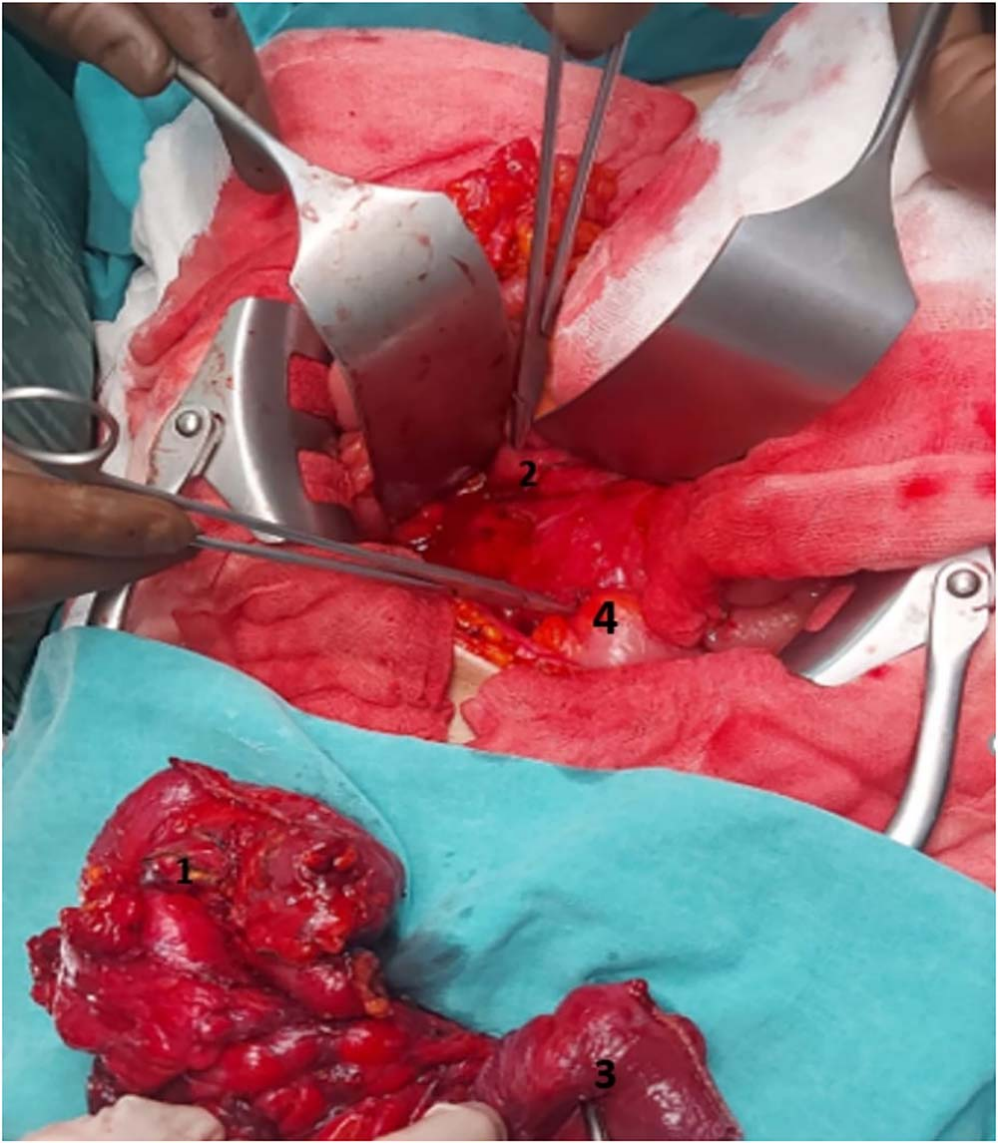
Operation area and surgical material. 2nd–3rd parts of the duodenum (2), sigmoid colon (4), terminal ileum (3), and cecum (1).

A 54-year-old male patient diagnosed with Crohn’s disease applied to our clinic with complaints of abdominal pain, diarrhea, and ~15 kg weight loss in the last year, which is a significant loss considering his initial weight of 65 kg. The patient had no history of surgery, but it was learned that he had been treated for miliary tuberculosis 2 years ago. Additionally, the patient is receiving medical treatment for Crohn’s disease. Upon examination, there was tenderness in the lower quadrants of the abdomen. Laboratory examinations revealed high C-reactive protein and white blood cell count. Abdominal ultrasonography showed wall thickening in the terminal ileum. Consequently, tomography enterography was performed 1 month after admission. Computed tomography (CT) showed thickening in the terminal ileum wall, an appearance suspicious for fistula between the duodenum and cecum and between the terminal ileum and sigmoid colon, and lymphadenopathy (Fig. 1). To further evaluate the suspected fistulae, colonoscopy was performed within 2 weeks of admission, and fistula orifices were identified in the sigmoid colon and terminal ileum. Following these findings, the patient was scheduled for surgery in the third month after presentation. During this period, the patient received oral antibiotic therapy to control a secondary infection. During the surgery, there were millimetric lesions in the small bowel mesentery that were suspicious for carcinomatosis, and a fistula appearance between the terminal ileum and sigmoid colon and between the cecum and the 2nd–3rd part of the duodenum. Frozen samples were taken from the millimetric lesions due to the preliminary diagnosis of carcinomatosis and were interpreted as benign. Thereupon, the patient underwent ileocecectomy, fistulectomy using linear stapler in the fistulized area in the duodenum and duodenal repair, and primary repair with vicryl in the fistulectomy area in the sigmoid colon (Fig. 2). Postoperatively, the patient was monitored in the intensive care unit for one day. Enteral nutrition was initiated gradually on the third postoperative day, starting with clear fluids and advancing to a soft diet. He was discharged on the 8th postoperative day without complications. Histopathological examination revealed ulcerated clonic mucosa, inflammatory granulation tissue, and fistula formation, consistent with Crohn’s disease. Additionally, biopsy samples were sent for mycobacterial culture to rule out tuberculosis, and the results were negative.

## Discussion

Crohn’s disease is a chronic inflammatory bowel disease characterized by inflammation and ulcerations that usually affects the small intestine and large intestine.

Diagnosis is usually made by clinical symptoms, laboratory tests, endoscopic findings and imaging methods. This case well illustrates the difficulties and complications in the diagnosis of Crohn’s disease. The literature frequently emphasizes that Crohn’s disease can present with various clinical findings and may be confused with infectious diseases [7].

Our patient presented with classic Crohn’s disease symptoms such as abdominal pain, diarrhea and weight loss, but the diagnostic process was initially complicated by his previous history of tuberculosis. Tuberculosis, especially the miliary form, can present with symptoms similar to those of digestive system diseases and can be confused with Crohn’s disease [8–11].

Studies suggest that the diagnosis of Crohn’s disease may be delayed, especially in patients with a history of active tuberculosis, and sometimes both conditions may coexist [12]. Therefore, differential diagnosis is of great importance in establishing the diagnosis of Crohn’s disease.

Imaging tests, particularly CT enterography, can help guide us. Single or isolated ileal focal lesions and necrosis or calcifications in the mesenteric lymph nodes are typical findings of intestinal Tuberculosis, while proximal and segmental involvement of the small intestine or the presence of fistulas should steer us towards Crohn’s disease [13].

In pathological evaluation, the presence of findings such as ulcers, inflammatory granulation tissue, and fistula formation confirms that our patient is consistent with Crohn’s disease. The literature emphasizes that fistula formation due to Crohn’s disease is frequently observed, but other conditions such as infections and malignancies can also lead to fistula development [14, 15].

In our patient, healing was rapid after surgical intervention, and no complications occurred. Surgical treatment plays an important role in controlling symptoms and improving the quality of life in patients with Crohn’s disease.

## Conclusion

This case is an important example of the clinical course, diagnosis, and treatment process of Crohn’s disease. In particular, the fact that Crohn’s disease and infectious diseases such as tuberculosis show similar symptoms can make the diagnosis process difficult. There is no distinct symptom pattern that can clearly distinguish between intestinal tuberculosis and Crohn’s disease [16, 17]. Common symptoms in Crohn’s disease patients typically include chronic diarrhea, abdominal pain, rectal bleeding, recurrent partial bowel obstructions, and perianal fistulas. On the other hand, intestinal Tuberculosis is commonly associated with fever, anorexia, weight loss, abdominal pain, changes in bowel habits, recurrent partial bowel obstructions, or an abdominal mass.

There is currently no gold standard method for distinguishing intestinal tuberculosis from Crohn’s disease, but the use of accurate diagnostic methods can improve diagnostic precision [18].

Such cases provide valuable lessons for clinical practice and the diagnostic process, once again highlighting the importance of a multidisciplinary approach.

## Data Availability

All data generated or analyzed during this study are included in this article. Further inquiries can be directed to the corresponding authors.

## References

[1] Tjandra D, Garg M, Behrenbruch C, et al. Review article: investigation and management of internal fistulae in Crohn's disease. Aliment Pharmacol Ther 2021;53:1064–79. 10.1111/apt.16326. 33721351

[2] Hellers G, Bergstrand O, Ewerth S, et al. Occurrence and outcome after primary treatment of anal fistulae in Crohn's disease. Gut 1980;21:525–7. 10.1136/gut.21.6.525. 7429313 PMC1419665

[3] Farmer RG, Hawk WA, Turnbull RB Jr. Clinical patterns in Crohn's disease: a statistical study of 615 cases. Gastroenterology 1975;68:627–35. 10.1016/S0016-5085(75)80270-8. 1123132

[4] Rankin GB, Watts HD, Melnyk CS, et al. National Cooperative Crohn's disease study: extraintestinal manifestations and perianal complications. Gastroenterology 1979;77:914–20. 10.1016/0016-5085(79)90391-3. 467943

[5] Iesalnieks I, Adamou A, Bercher M. P489 surgery for enterocutaneous fistula: the strongest predictor of postoperative mortality in Crohn’s disease patients. J Crohn's Colitis 2023;17:i618–9. 10.1093/ecco-jcc/jjac190.0619.

[6] Kaimakliotis P, Simillis C, Harbord M, et al. A systematic review assessing medical treatment for rectovaginal and enterovesical fistulae in Crohn's disease. J Clin Gastroenterol 2016;50:714–21. 10.1097/MCG.0000000000000607. 27466166

[7] De Hertogh G, Geboes K. Crohn's disease and infections: a complex relationship. MedGenMed 2004;6:14. PMC1435589 15520637

[8] Huang X, Liao WD, Yu C, et al. Differences in clinical features of Crohn's disease and intestinal tuberculosis. World J Gastroenterol 2015;21:3650–6. 10.3748/wjg.v21.i12.3650. 25834333 PMC4375590

[9] Onal IK, Kekilli M, Tanoglu A, et al. Tuberculosis and Crohn's disease revisited. J Coll Physicians Surg Pak 2015;25:443–8. 26100999

[10] Navaneethan U, Cherian JV, Prabhu R, et al. Distinguishing tuberculosis and Crohn's disease in developing countries: how certain can you be of the diagnosis? Saudi J Gastroenterol 2009;15:142–4. 10.4103/1319-3767.49012. 19568588 PMC2702967

[11] Patel N, Amarapurkar D, Agal S, et al. Gastrointestinal luminal tuberculosis: establishing the diagnosis. J Gastroenterol Hepatol 2004;19:1240–6. 10.1111/j.1440-1746.2004.03485.x. 15482529

[12] Rolo R, Campainha S, Duarte R. Crohn's disease and intestinal tuberculosis: a clinical challenge. Rev Port Pneumol 2012;18:205–6. 10.1016/j.rppneu.2012.02.006. 22405954

[13] Mao R, Liao WD, He Y, et al. Computed tomographic enterography adds value to colonoscopy in differentiating Crohn’s disease from intestinal tuberculosis: a potential diagnostic algorithm. Endoscopy 2015;47:322–9. 10.1055/s-0034-1391230. 25675175

[14] Sunday-Adeoye I, Eni UE, Ekwedigwe KC, et al. Enterocutaneous fistula coexisting with enterovesical fistula: a rare complication of ovarian cystectomy. Afr J Reprod Health 2019;23:139–49. 10.29063/ajrh2019/v23i1.15. 31034181

[15] Mosquera-Klinger G, Torres-Rincón R, Jaime-Carvajal J. Endoscopic closure of gastrointestinal perforations and fistulas using the Ovesco over-the-scope clip system at a tertiary care hospital center. Rev Gastroenterol Mex (Engl Ed) 2019;84:263–6. 10.1016/j.rgmx.2018.10.004. 31014750

[16] Sood A, Midha V, Singh A. Differential diagnosis of Crohn's disease versus ileal tuberculosis. Curr Gastroenterol Rep 2014;16:418. 10.1007/s11894-014-0418-9. 25277043

[17] Amarapurkar DN, Patel ND, Rane PS. Diagnosis of Crohn's disease in India where tuberculosis is widely prevalent. World J Gastroenterol 2008;14:741–6. 10.3748/wjg.14.741. 18205265 PMC2684002

[18] Uygur-Bayramicli O, Dabak G, Dabak R. A clinical dilemma: abdominal tuberculosis. World J Gastroenterol 2003;9:1098–101. 10.3748/wjg.v9.i5.1098. 12717865 PMC4611381

